# Potential role of FoxO3a in the regulation of trophoblast development and pregnancy complications

**DOI:** 10.1111/jcmm.16499

**Published:** 2021-04-03

**Authors:** Hao Chen, Xin Tang, Ting‐Li Han, Jia‐Nan Zhu, Wei Zhou, Philip N. Baker, Chang Chen, Hua Zhang

**Affiliations:** ^1^ Department of Obstetrics and Gynecology The First Affiliated Hospital of Chongqing Medical University Chongqing China; ^2^ Canada‐China‐New Zealand Joint Laboratory of Maternal and Fetal Medicine Chongqing Medical University Chongqing China; ^3^ The Chongqing Key Laboratory of Translational Medicine in Major Metabolic Diseases Chongqing China; ^4^ Department of Obstetrics and Gynecology The Second Affiliated Hospital of Chongqing Medical University Chongqing China; ^5^ Department of Obstetrics Chongqing Health Center for Women and Children Chongqing China; ^6^ College of Medicine, Biological Sciences and Psychology University of Leicester Leicester UK; ^7^ Institute of Life Sciences Chongqing Medical University Chongqing China

**Keywords:** forkhead box O3a protein, intercellular cell adhesion molecule‐1, migration, pre‐eclampsia, transcriptomics

## Abstract

The forkhead box O3a protein (FoxO3a) has been reported to regulate tumour invasion and migration, but little is known about the molecular mechanism or its role in trophoblast invasion and migration into the uterus. In this study, we aim to explore its role in trophoblast development and placenta‐related pregnancy complications and the potential mechanism. Levels of FoxO3a and its phosphorylated form (p‐FoxO3a) in placental tissue from healthy pregnant women and pre‐eclampsia patients were first compared. Then, HTR‐8/SVneo cells were transfected with lentiviral vectors to deplete and overexpress FoxO3a. Western blot, immunohistochemistry, Cell Counting Kit‐8, wound‐healing assay, Matrigel invasion assay, cell apoptosis, cell cycle assay, RNA sequencing, qRT‐PCR and ChIP‐qPCR were performed on the cells to study the potential role of FoxO3a and the underlying mechanism. We found the expression of FoxO3a was decreased, whereas p‐FoxO3a was increased in pre‐eclampsia placentae. FoxO3a depletion significantly reduced transcription of the promoter region of intercellular cell adhesion molecule‐1 (ICAM1) gene in ChIP assays and led to reduced invasion and migration of trophoblast cells, arrested cell cycle in G1 phase and increased apoptosis under oxidative stress. Our results suggested that FoxO3a may play a role in the regulation of trophoblast invasion and migration during placental development, which may be because of its affinity to the ICAM1 promotor.

## 1 INTRODUCTION

The placenta is the intermediary organ that connects the mother and foetus during pregnancy. Its development started with trophoblast invasion and migration into the uterus.^1^, ^2^, ^3^ Dysfunction of trophoblast cell results in superficial invasion and insufficient remodelling of spiral uterine artery, which ultimately leads to placental dysplasia and pregnancy complications including pre‐eclampsia, foetal growth restriction, still­birth and preterm birth.^4^, ^5^, ^6^, ^7^ Therefore, the invasion and migration capacity of trophoblast is worthy of attention when investigating the aetiology of the placenta‐related pregnancy complications.

The invasion, migration, apoptosis and differentiation of trophoblast cells are regulated by different transcription factors and signalling pathways.^8^, ^9^, ^10^, ^11^ The forkhead box O3a (FoxO3a) is an important member of the O subfamily of the forkhead protein transcription factor family (Foxes).^12^ It is expressed in various organs and tissues such as placenta, heart, vascular endothelium and fat and is involved in biological processes such as cell development, metabolism, apoptosis, autophagy and anti‐oxidative stress.^13^, ^14^, ^15^, ^16^ The function of FoxO3a in tumour cells has been studied previously. When the phosphoinositide 3‐kinase (PI3K) and protein kinase B (Akt) are activated, FoxO3a is phosphorylated and transferred from the nucleus to the cytoplasm, thus reducing the FoxO3a level in the nucleus and the expression of downstream genes promoted by FoxO3a.^17^, ^18^ The decreased FoxO3a expression in tumour cells was reported to be associated with decreased MMP1, MMP9 and MMP13 levels and inhibited invasion and migration.^19^, ^20^, ^21^ In another study, FoxO3a activated by PI3K or AKT inhibitors acted like a metastasis inducer.^22^ However, the molecular mechanism of how FoxO3a promotes the expression of downstream genes has not been clarified yet. Besides, the behaviour of trophoblasts is somewhat similar to tumour cells,^23^ in that both types of cells invade to adjacent tissue, with accelerated metabolism and cell cycle. Although studies have shown that FoxO3a plays an important regulatory role in tumours, its effects on trophoblast migration, invasion, metabolism, cell cycle and apoptosis are unknown.

Hence, this study aims to investigate FoxO3a in HTR8/neo cell line (a well‐recognized cell line similar to human trophoblast cells) and the related mechanism, with the hope of understanding its role and related mechanism in placental development and placenta‐related pregnancy complications.

## 2 METHODS AND MATERIALS

### 2.1 Human placenta samples

Placental sample collection was approved by the ethical committee of The First Affiliated Hospital of Chongqing Medical University and with the informed consent of each patient. The study conforms to the Ethical Review Methods for Biomedical Research involving Humans adopted by the National Health and Family Planning Commission of the People's Republic of China. Women with severe pre‐eclampsia (PE, n = 7) and age‐matched normotensive pregnancies (NC, n = 7) underwent elective caesarean section at the Department of Obstetrics were randomly recruited into this study. The diagnosis of severe pre‐eclampsia was based on the guidelines of the American College of Obstetrics and Gynecology (ACOG).^24^ The exclusion criteria included diabetes, chronic hypertension, immune diseases, infections and pregnancy complications other than pre‐eclampsia. All the women in the study had singleton pregnancy. The clinical characteristics of the participants are shown in Table 1.

**TABLE 1:** Clinical characteristics of studied pregnant women

Category	sPE^a^(n = 7)	Normal(n = 7)	*P*–value
Age (years)	31.0 ± 3.4	31.9 ± 6.8	0.88
BMI at delivery (kg/m^2^)	29.4 ± 3.1	26.1 ± 2.3	0.67
Gestational age at delivery (weeks)	38.7 ± 1.5	35.1 ± 0.5	0.88
Parity	0.3 ± 0.5	1.1 ± 0.6	0.67
Systolic blood pressure (mmHg)	173.7 ± 9.7	110.1 ± 4.9	<0.000001
Diastolic blood pressure (mmHg)	111.4 ± 8.5	68.0 ± 4.5	<0.000001
Proteinuria	3.0 ± 0.8	0.0 ± 0.0	0.000003
Placental weight(g)	608.6 ± 59.4	648.9 ± 124.3	0.089474
Neonatal birth weight (g)	3141.4 ± 303.1	3058.6 ± 587.5	0.88

^a^ Severe pre‐eclampsia.

Placental tissue was collected, soaked in iced saline and quickly transferred to the laboratory for further preparation. The samples were cut into small pieces and washed with iced PBS for three times. A proportion of the specimen were frozen at −80°C for Western blot analysis, and the remaining was embedded with paraffin for immunohistochemistry analysis.

### 2.2 Cell culture and treatment

Human trophoblast HTR‐8/SVneo cell line was obtained from the American Type Culture Collection (ATCC) and cultured in RPMI 1640 medium with L‐Glutamine (Gibco), 10% foetal bovine serum (FBS, PAN, Germany) and 1% penicillin and streptomycin. The cells were grown in standard culturing conditions (37°C and humidified atmosphere with 5% CO_2_). Cells were transfected with lentiviral vectors (GenePharma) for 48 hours and divided into three groups: control group (shNC), scrambled shRNA group (shFoxO3a) and overexpression group (oeFoxo3a). Sodium nitroprusside (SNP) was used to simulate oxidative stress.

### 2.3 Immunohistochemistry

Paraffin‐embedded placental tissue was cut into 4μm‐thick slices. The tissue slices were dewaxed, hydrated with gradient ethanol and washed. After antigen retrieval with saline sodium citrate, H_2_O_2_ was used to block endogenous peroxides. Then, the corresponding primary antibody for FoxO3a (1:200, Catalog#:12 829, Cell Signaling Technology), cytokeratin 7 (1:200, Catalog#: ab68459, Abcam) or human leucocyte antigen‐G (1:200, Catalog#: ab52455, Abcam) was added and incubated overnight. After reheating, the reaction enhancer solution and sheep anti‐mouse/rabbit IgG (ZSGB‐BIO) was added. The signals were displayed with diaminobenzidine (DAB staining, ZSGB‐BIO).

### 2.4 Western blot

Placental and cellular protein was extracted using RIPA lysis buffer (Beyotime Biotechnology, China) with PMSF (1:100, Beyotime Biotechnology). Protein concentration after centrifugation was measured with BCA Protein Assay Kit (Beyotime Biotechnology, China). Each protein sample was transferred to a PVDF membrane after electrophoresis on 10% SDS‐PAGE. The membranes were blocked with TBST containing 5% skimmed milk powder for 1 hour and then incubated with corresponding primary rabbit polyclonal antibodies, including anti‐FoxO3a (1:800, Catalog#:12829, Cell Signaling Technology), p‐FoxO3a (1:1000, Catalog#: ab154786, Abcam) and β‐actin (1:5000, Catalog#: GB11001, Servicebio), at 4°C overnight.

### 2.5 Cell proliferation assay

Cell Counting Kit‐8 (CCK‐8, Beyotime) was used to detect cell proliferation. Approximately 5 × 10^3^ HTR8/SVneo cells were seeded in the central part of each well in a 96‐well plate. At 24, 48 and 72h after seeding, 10 μL CCK‐8 solution was added into each well and incubated for 2h. A microplate reader (Thermo Fisher Scientific) was used to measure the absorbance at 450 nm for each well. Each experiment was performed in triplicate.

### 2.6 Cell migration assay

Wound‐healing assay was used to evaluate the migration of HTR8‐S/Vneo cells. 5 × 10^5^ cells were seeded into each well of a 6‐well plate. When the cells reached 90% confluence, the cells were scratched with a 200 µL pipette tip and washed with PBS. Images were taken at 0h and 24h after the scratch. The wound‐healing rate was measured with ImageJ software. Each experiment was performed in triplicate.

### 2.7 Cell invasion assay

The invasiveness of the HTR8/SVneo cells were detected using Matrigel invasion assay. Four hours before seeding the cells, Matrigel (1:5 dilution, BD BioScience) was spread in each chamber. Then, approximately 5 × 10^4^ cells were seeded into each Matrigel invasion chamber placed in 24‐well plates. After incubation for 24 hours, the cells were wiped from the upper chamber using a cotton swab. The cells in the lower chamber were fixed with 4% paraformaldehyde and stained by crystal violet (Beyotime Biotechnology). The lower chamber was then photographed under a microscopy (EVOS FL Auto Imaging System, Life Technologies). The invasion rate was measured with ImageJ software. Each experiment was performed in triplicate.

### 2.8 Cell apoptosis and cell cycle assay

The three groups of HTR8/SVneo cells, treated with or without SNP, were collected in the logarithmic growth phase. Cells digested with trypsin were washed twice with PBS and suspended in 500 μL PBS for apoptosis assay, or in 100 μL PBS followed by addition of 500 μL iced 75% ethanol for cell cycle assay. A flow cytometer (CytoFLEX, eBioscience) was used to analyse the samples for apoptosis and cell cycle according to the manufacturer's instructions.

### 2.9 RNA sequencing

HTR8‐S/Vneo cells were collected when grown to 80%‐90% fusion. Total RNA was extracted with TRIzol (Invitrogen) according to the manufacturer's protocol. Then, the quantity of RNA was measured by an Agilent 2100 Bioanalyzer (Agilent Technologies) and detected using RNase free agarose gel electrophoresis. mRNA was enriched with Oligo(dT) beads after total RNA extraction. The enriched RNA fragments were then smashed, and cDNA was synthesized with random primers. The cDNA was then purified using QiaQuick PCR extraction kit (Qiagen), end repaired, poly(A) added and connected to Illumina sequencing adapters. The ligation products were size selected by agarose gel electrophoresis, PCR amplified and sequenced using Illumina HiSeq2500 by Gene Denovo Biotechnology Co. DESeq2 software was used for RNA differential expression analysis^25^ between two groups (and by edgeR^26^ between two samples). The transcripts with false discovery rate (FDR) below 0.05 and absolute fold change ≥2 were considered differently expressed transcripts. GO Enrichment Analysis, Disease Ontology Enrichment Analysis, Pathway Enrichment Analysis and Protein‐Protein interaction Gene Set Enrichment Analysis (GESA) were applied to these differently expressed genes.

### 2.10 qRT‐PCR

RNA was isolated from the cultured cell lines by TRIzol reagent (Invitrogen) according to the manufacturer's instructions. The concentration of RNA was measured using ultraviolet spectroscopy (Nano Drop 2000, Thermo). Subsequently, the RNA was reversely transcribed to cDNA using Roche Reverse Transcription Kit (#07912455001, Roche). GAPDH was used as an endogenous control for gene expression analysis. Its primer pairs were as follows: forward: 5′ GGAAGCTTGTCATCAATGGAAATC 3′, reverse: 5′ TGATGACCCTTTTGGCTCCC 3′. The primer pairs of the tested genes were as follows: ICAM1: forward: 5′ CCGTTGCCTAAAAAGGAGTTGC 3′, reverse: 5′ TGATGACCCTTTTGGCTCCC 3′; Vcam1: forward: 5′AGGCTGGAAGAAGCAGAAAGGA 3′, reverse: 5′ ACTGGGCCTTTCGGATGGTAT 3′; ptgs2: forward: 5′AAGACAGATCATAAGCGAGGGC 3′, reverse: 5′ AAACCGTAGATGCTCAGGGACT 3′; MMP1: forward: 5′GGACCATGCCATTGAGAAAGC 3′, reverse: 5′ TTGTCCCGATGATCTCCCCT 3′; MMP9: forward: 5′TCGACGTGAAGGCGCAGAT 3′, reverse: 5′ AGAAGCGGTCCTGGCAGAAATA 3′, cyclin D1: forward: 5′GCTGGAGCCCGTGAAAAAG 3′, reverse: 5′ ACAGAGGGCAACGAAGGTC 3′.

### 2.11 ChIP‐qPCR

Chromatin immunoprecipitation (ChIP) assays were performed with EZ‐Zyme™ Chromatin Prep Kit (#17‐375, Millipore) and EZ‐Magna ChIP™ HiSens Chromatin Immunoprecipitation Kit (#1710461, Millipore) according to the manufacturer's protocols. The antibodies used for ChIP assay were anti‐FoxO3a (Catalog#:12 829, Cell Signaling Technology, 1 μg/test) and control IgG (#CS200581, Millipore, 1 μg/test). Real‐time PCR was performed to quantify the precipitated DNA samples. Data are shown as the expression percentages of input DNA. The primer pairs of ICAM1 were as follows: forward: 5' GACGTGGTGGATGTCGAGTCT 3', reverse: 5' TGCCTGTCGCTGGGATA 3'.

## 3 RESULTS

### 3.1 Lower expression of FoxO3a was found in pre‐eclampsia placenta

**FIGURE 1:**
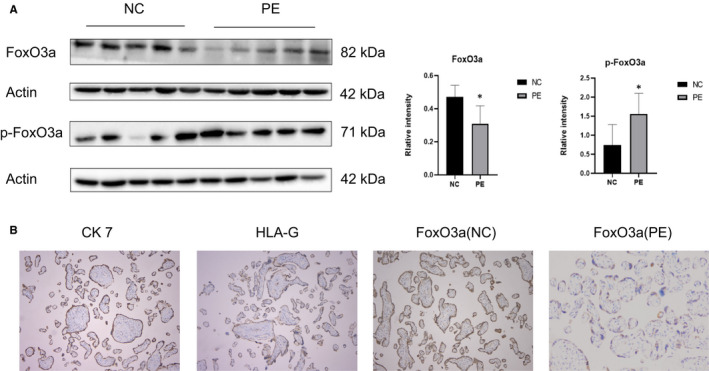
Expression and distribution of FoxO3a in placental tissues. (A) Western blot analysis of FoxO3a and p‐FoxO3a. (B) CK7 and HLA‐G were markers for trophoblast cells. The distribution of FoxO3a in women with normal or pre‐eclamptic pregnancies was localized in trophoblast cells (200×; scale bar, 200 μm). Results are shown as mean ± SEM, n = 5, *P < .05. NC: Normal control, PE: pre‐eclampsia

The expression of FoxO3a in the placenta of pregnant women with pre‐eclampsia was significantly lower than those with normal pregnancy, whereas the expression of phosphorylated FoxO3a was remarkably higher in pre‐eclampsia (Figure 1A). The immunohistochemistry results revealed that FoxO3a was present in trophoblast, which also expresses CK7 and HLA‐G (Figure 1B).

### 3.2 FoxO3a was knocked down and overexpressed in HTR8‐S/Vneo cells

**FIGURE 2:**
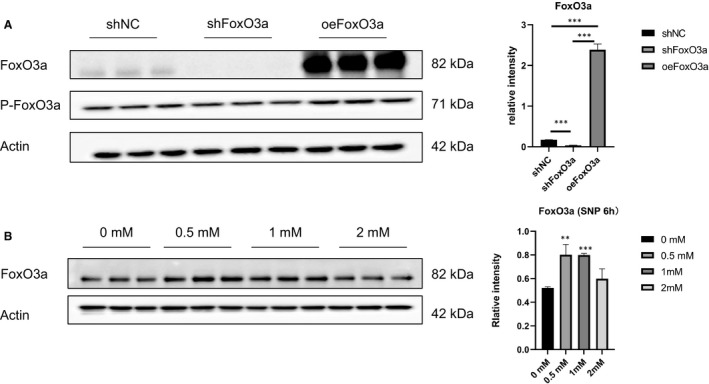
Transfection efficiency of lentivirus targeting FoxO3a in HTR8/SVneo cells and the level of FoxO3a in HTR8/SVneo cells treated with SNP at different concentrations. (A) Western blot analyses of FoxO3a for transfection efficiency of lentivirus after the HTR8/SVneo cells were transfected for 48 hours. (B) Western blot analyses of FoxO3a after treatment with SNP at increasing concentrations for 6 hours. Results are shown as mean ±SEM, n = 3, **P < .01 and ***P < .001

To explore the role of FoxO3a in pre‐eclampsia, the FoxO3a gene was overexpressed or knocked down through lentiviral transfection. Compared with the control group, the expression of FoxO3a was significantly down‐regulated in the knockdown group and up‐regulated in the overexpression group (Figure 2A). Various concentrations of SNP could also induce the expression of FoxO3a in HTR8/SVneo cells, among which 0.5 mM showed the most significant effect (Figure 2B).

### 3.3 FoxO3a regulates ICAM1 transcription

**FIGURE 3:**
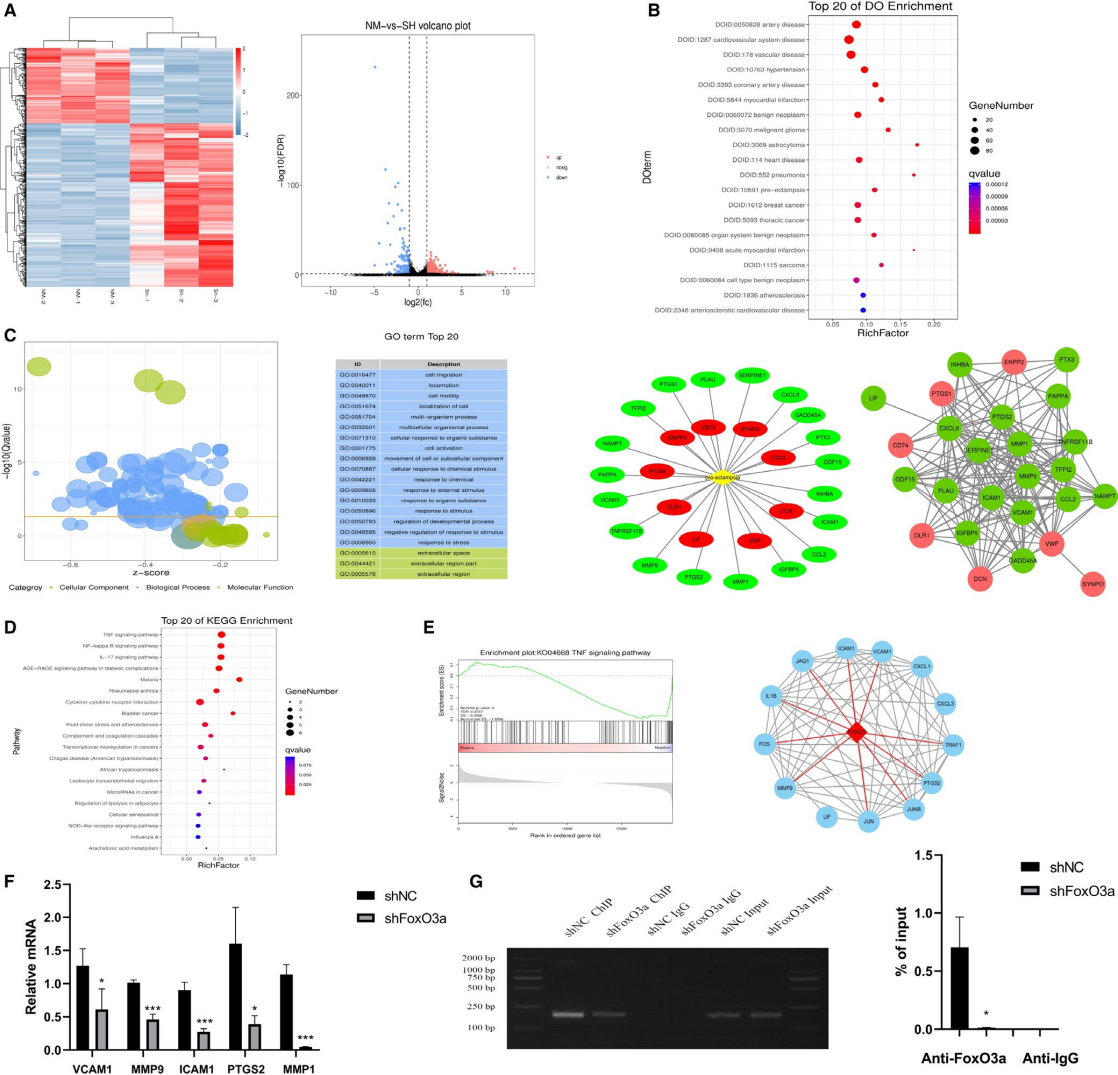
FoxO3a regulates gene expression of HTR8/SVneo cells and ICAM1 transcription. (A) The heat map and Volcano plot illustrate the copy numbers of mRNAs. Red represents higher transcription, and blue represents lower transcription. (B) Disease ontology analysis shows 28 genes relate to pre‐eclampsia. (C) Enriched Gene Ontology (GO) analysis shows migration is the most affected biological process after FoxO3a knockdown in HTR8/SVneo cells. (D) KEGG pathway analysis shows the related pathways. (E) GSEA and protein‐protein interaction (PPI) analysis shows the decreased TNF signalling pathway. (F) qPCR shows reduced expression of the genes downstream of TNF signalling pathway. (G) ChIP‐PCR and ChIP‐qPCR for FoxO3a indicate it binds to the promoter region of ICAM1. Results are shown as mean ±SEM, n = 3, *P < .05, ***P < .001

To explore the molecular mechanism related to FoxO3a, we performed RNA‐seq test and discovered 469 differential genes in the shFoxO3a group, including 321 up‐regulated ones and 148 down‐regulated ones (Figure 3A). Disease ontology analysis revealed that 28 out of these 469 genes were related to pre‐eclampsia (Figure 3B). Gene ontology analysis of the 469 genes showed that cell migration was the most affected biological process (Figure 3C). KEGG pathway analysis unveiled that FoxO3a was related to a number of signalling pathways, such as TNF, NF‐kappa B, IL‐17 and AGE‐RAGE (Figure 3D). Protein‐protein interaction (PPI) analysis found that FoxO3a was directly related to genes downstream of the TNF signalling pathway (Figure 3E). Gene set enrichment analysis confirmed that TNF signalling pathway was down‐regulated after FoxO3a depletion (Figure 3E). qPCR unveiled that the expression of downstream genes of TNF signalling pathway, including intercellular cell adhesion molecule‐1 (ICAM1), vascular cell adhesion molecule (VCAM1), prostaglandin‐endoperoxide synthase 2 (PTGS2), MMP9 and MMP1, was decreased (Figure 3F).

Notably, we found that the transcription of ICAM1 was significantly decreased (*P* < .001) through RNA‐seq data mining. To verify the finding, we further performed ChIP‐PCR and ChIP‐qPCR assay on the promoter region of ICAM1 and found the enrichment of Foxo3a at the ICAM1 promoter was significantly reduced after FoxO3a depletion (Figure 3G).

### 3.4 FoxO3a knockdown and overexpression affected apoptosis, cell cycle and proliferation of HTR8/SVneo cells

**FIGURE 4:**
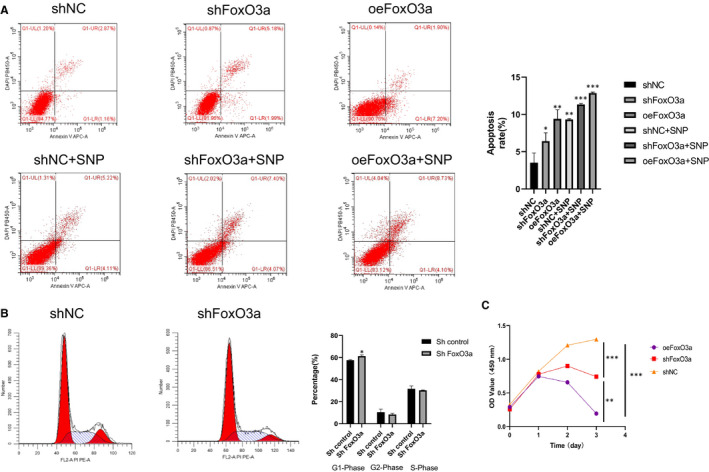
The change of apoptosis, cell cycle and proliferation of HTR8/SVneo cells with FoxO3a knockdown and overexpression. (A) Apoptosis of HTR8‐S/Vneo cells was detected by flow cytometry. (B) The alterations of cell cycle of HTR8‐S/Vneo cells were detected by flow cytometry. (C) CCK‐8 analysis was used to test proliferation of HTR8‐S/Vneo cells. Results are shown as mean ±SEM, n = 3, *P < .05, **P < .01 and ***P < .001

The consequences of FoxO3a knockdown and overexpression on HTR8‐S/Vneo cells were not simply inverse. Cell cycle and apoptosis alterations of HTR8‐S/Vneo cells were detected by flow cytometry. The apoptotic rates increased in both the shFoxO3a group and oeFoxO3a group compared with the shNC group (Figure 4A), with the increase in the oeFoxO3a group being more apparent. In regard to the cell cycle, the HTR8‐S/Vneo cell in the shFoxO3a group showed a rise in phase G1 ratio and decline in phase G2 compared with the shNC group (Figure 4B). Proliferation of HTR8/SVneo cells was detected by CCK‐8 assay. Compared with the shNC group, cell proliferation rates fell in both the shFoxO3a group and oeFoxO3a group, and the latter was more remarkable (Figure 4C).

### 3.5 FoxO3a knockdown restrained the migration and invasion of HTR8/SVneo cells

**FIGURE 5:**
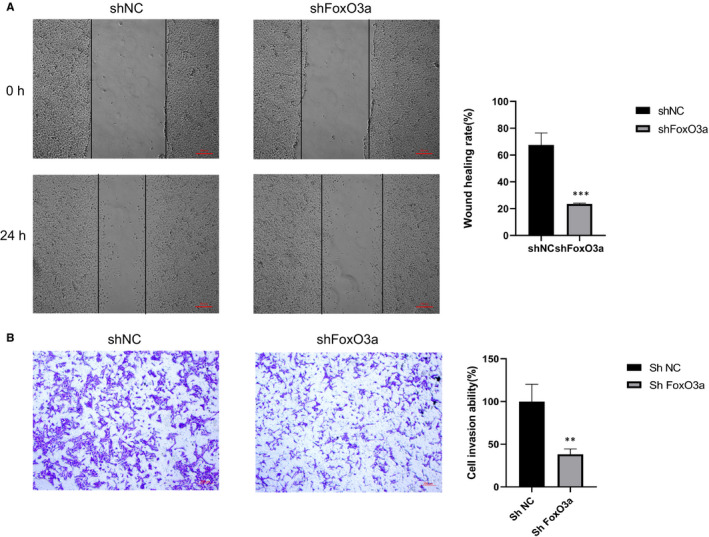
The change of migration and invasion of HTR8/SVneo cells after FoxO3a knockdown. (A) Wound‐healing assay, n = 3 for each group, scale bars: 200 μm; and (B) Matrigel transwell assay, n = 3 for each group. Results are shown as mean ±SEM, **P < .01, ***P < .001

We used Wound‐healing assay and Matrigel cell invasion assay to investigate the effect of FoxO3a knockdown on HTR8/SVneo cells. It was found that the wound‐healing rate (Figure 5A) and Matrigel cell invasion (Figure 5B) of the shFoxO3a group were significantly decreased compared with the shNC group.

## 4 DISCUSSION

The placental implantation and vascular recasting occur in the first trimester. For ethical reasons, we could not collect clinical samples during that period and hence used placenta at late pregnancy and HTR‐8/SVneo cell line to study the regulatory role of FoxO3a in placental development. Our data showed lower expression of FoxO3a in pre‐eclampsia placenta than in normal ones, and depletion of FoxO3a in HTR8/SVneo cell reduced its migration and invasion. Both knockdown and overexpression of FoxO3a promoted its apoptosis.

In order to investigate how FoxO3a affecting trophoblast migration, invasion, metabolism, cell cycle and apoptosis, we conducted RNA‐seq and following bioinformatics analysis. The results showed that FoxO3a was mainly involved in the migration of cells. The genes ICAM1, VCAM1, PTGS2, MMP1 and MMP9, as well as the signalling pathways TNF, NF‐kappa B, IL‐17 and AGE‐RAGE, may be associated with this process. It has been reported that ICAM1, VCAM1, PTGS2, MMP9 and MMP1 restrain migration, invasion^19^, ^20^, ^21^ and promote apoptosis^27^, ^28^ in tumour cells, which is in agreement with this study.

Notably, the qRT‐PCR results suggested that FoxO3a may be associated with ICAM1. ICAM1 is a member of the immunoglobulin superfamily. It is the ligand for the b2 integrin lymphocyte function‐associated antigen‐1 and CD11b/CD18. ICAM1 is expressed in a variety of haematopoietic and non‐haematopoietic cells, including B and T cells, macrophages, dendritic cells, fibroblasts, keratinocytes, endothelial cells, and certain epithelial tissues.^29^, ^30^ A large number of previous studies have shown that ICAM1 is highly expressed in many tissues such as placenta, bone marrow, periodontal ligament and fat.^31^, ^32^, ^33^ ICAM1 also plays an important role in the development of different carcinomas, and its reduction suppresses the metastasis of cancers such as liver,^34^ breast,^35^ colorectal ^36^ and lung cancer.^37^ So, it is a target of some anti‐cancer drugs. To further explore the relation between FoxO3a and ICAM1, we performed Chip‐PCR and Chip‐qPCR experiments. Interestingly, we found that FoxO3a directly regulated the expression of ICAM1, and the enrichment of ICAM1 was significantly reduced after FoxO3a depletion (Figure 3G). These results were consistent with previous studies showing that ICAM1 plays an antiapoptotic effect and resists Fas‐induced chondrocyte apoptosis.^27^ In another report, ICAM1 was shown to be involved in signalling pathways that affect cell proliferation and apoptosis, and its expression contributes to antiapoptotic effects.^28^ In addition, increased expression of ICAM1 promotes tumour development by enhancing cancer cell invasion and migration ability.^31^, ^32^, ^33^

It is known that FoxO3a plays an essential role on cell cycle. In this study (Figure 4), we also observed that higher ratios of HTR8/SVneo cells transfected with shFoxO3a lentiviral remained in G1/G0 phase, and cell proliferation was restrained. Also, we observed that the migration and invasion of HTR8/SVneo cells with FoxO3a knockdown were suppressed (Figure 5), whereas apoptosis increased (Figure 4A). Interestingly, the apoptosis rate of cells with overexpression of FoxO3a was significantly increased. These results may be attributed to the genes downstream of FoxO3a and related to apoptosis, including Bim, Fasl, TRAIL and Bcl.^38^, ^39^, ^40^, ^41^, ^42^

In conclusion, our results found that FoxO3a plays an important role in the apoptosis, migration and invasion of HTR8‐S/Vneo cells, which may be partially explained by its affinity to the ICAM1 promotor.

## CONFLICT OF INTERESTS

The authors indicate that there is no potential conflict of interest.

## AUTHOR CONTRIBUTIONS

**Hao Chen:** Investigation (equal); Writing‐original draft (equal). **Xin Tang:** Data curation (equal); Formal analysis (equal); Investigation (equal). **Ting‐Li Han:** Methodology (lead); Software (lead); Visualization (lead); Writing‐review & editing (supporting). **Jia‐Nan Zhu:** Investigation (supporting). **Wei Zhou:** Investigation (supporting). **Philip N. Baker:** Conceptualization (equal); Project administration (equal). **Chang Chen:** Formal analysis (supporting); Writing‐original draft (equal); Writing‐review & editing (lead). **Hua Zhang:** Conceptualization (equal); Project administration (lead); Resources (lead); Supervision (lead).

## Data Availability

The data that support the findings of this study are available from the corresponding author upon reasonable request.
